# Levistilide A Ameliorates NLRP3 Expression Involving the Syk-p38/JNK Pathway and Peripheral Obliterans in Rats

**DOI:** 10.1155/2018/7304096

**Published:** 2018-08-12

**Authors:** Huining Guo, Li Sun, Shuang Ling, Jin-Wen Xu

**Affiliations:** Institute of Interdisciplinary Medical Science, Shanghai University of Traditional Chinese Medicine, Shanghai 201203, China

## Abstract

**Background:**

Inflammation is one of the most important pathogeneses of thromboangiitis obliterans (TAO). The NLRP3 inflammasome plays a vital role in the body's immune response and disease development. It can be activated by numerous types of pathogens or danger signals. As the core of the inflammatory response, the NLRP3 inflammasome may provide a new target for the treatment of various inflammatory diseases. Levistilide A (LA) is a phthalide dimer isolated from umbelliferous plants. Its pharmacological effect is largely unknown. This study revealed the effects of LA on endothelial cell activation, NLRP3, IL-1*β*, TNF-*α*, IL-32, and CCL-2, VCAM-1, MCP-1, and the spleen tyrosine kinase (Syk)--p38/JNK signaling axis and its effect on vasculitis in rats.

**Results:**

LA inhibited endothelial activation and the expression of IL-1*β*, TNF-*α*, IL-32, CCL-2, VCAM-1, and MCP-1. LA directly obstructed Syk phosphorylation and activity in a dose-dependent manner, inhibited the activity of p38 and JNK, and reduced the expression of NLRP3 in human umbilical vein endothelial cells and vascular tissue of rats with vasculitis.

**Conclusion:**

LA suppressed NLRP3 gene expression by blocking the Syk--p38/JNK pathway and reduced damage to the rats' limbs in the thromboangiitis obliterans model.

## 1. Introduction

Vasculitis is an inflammatory cell infiltration around blood vessel walls and blood vessels, accompanied by vascular damage, cellulose deposition, fibrosis, and endothelial cell and myocyte necrosis. Vasculitis is characterized by systemic inflammation and endothelial injury [1]. Endothelial activation and injury are fundamental to the pathogenesis. Endothelial activation and injury increase expression of endothelial cell adhesion and cause endothelial switching to the prothrombotic phenotype. Thromboangiitis obliterans (TAO) or Buerger's disease (BD) is a thrombotic occlusive, nonatherosclerotic segmental inflammatory disease that causes ischemia in small and medium vessels in the extremities of the limbs. TAO is characterized by segmental thrombotic occlusions by highly mononuclear cellular thrombi. Endothelial dysfunction may play a crucial role in BD [2]. Patients with BD have a diminished capability for endothelium-dependent vasodilation and higher levels of some circulating markers of inflammation, such as leukocytes, C-reactive protein, and intercellular adhesion molecule-1 [3].

**Figure 1:**
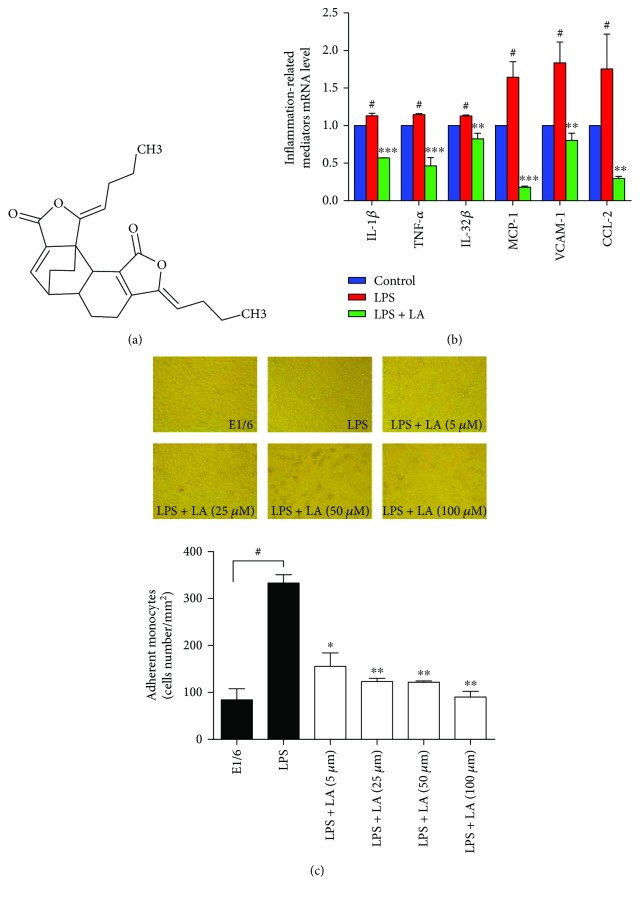
LA inhibited the mRNA levels of LPS-induced proinflammatory mediators and inhibited the adhesion of monocytes to HUVECs. The molecular structure of LA (a). HUVECs were pretreated with 50 *μ*M of LA for 60 min before treatment with LPS (100 ng/mL) (b) for 6 h. Expression of IL-1*β*, TNF-*α*, IL-32*β*, MCP-1, CCL-2, and VCAM-1 mRNA levels were measured using RT-PCR. HUVECs were pretreated with a blocking mouse antihuman monoclonal antibody (E1/6) against VCAM-1 or various doses of LA for 60 min before treatment with LPS. U937 mononuclear cells adhered to LPS-stimulated HUVECs were observed through microscopic images (c). Data represent the mean ± SD of at least three independent experiments, and each experiment was performed in triplicate. ^#^*p* < 0.05 versus control; ^∗^*p* < 0.05 versus LPS; ^∗∗^*p* < 0.01 versus LPS; ^∗∗∗^*p* < 0.001 versus LPS.

Levistilide A (LA) (Figure 1(a)) is a phthalide dimer isolated from *Ligusticum chuanxiong* and *Angelica sinensis*, both of them belonging to the Umbelliferae herb family [4–7]. These herbs are often used in oriental traditional medicinal formulas, such as Danggui-Shaoyao-San (DSS). At present, there are few reports on LA research. LA inhibits PDGF-BB-activated hepatic stellate cell proliferation; the mechanism may involve cell cycle inhibition and apoptosis mechanisms. LA might be a potential antifibrotic drug for the treatment and prevention of hepatic fibrosis [8]. LA can also induce apoptosis of colon cancer cells through a reactive oxygen species- (ROS-) mediated endoplasmic reticulum (ER) stress pathway [9]. He et al. administered LA orally to rats and measured some major pharmacokinetic parameters; the rate of elimination is 0.183 ± 0.02 h^−1^, the rate of absorption is 0.781 ± 0.08 h^−1^, the rate of distribution is 0.815 ± 0.06 h^−1^, *t*_1/2*β*_ is 3.789 ± 0.38 h, *t*_1/2a_ is 0.857 ± 0.09 h, *T*_max_ is 0.5 ± 0.05 h, and *C*_max_ is 1149.2 ± 103.43 ng/mL [10].

Xiong et al. proved that LA is an anti-inflammatory quality marker of Xiaojin pills [11]; Xiong et al.'s paper may be the only report describing LA's anti-inflammatory effects. However, the research about LA's anti-inflammatory biological mechanism has not yet been reported. Choi et al. found that ligustilide can attenuate vascular inflammation [12]. LA is a dimer of ligustilide; one can speculate that some connection may exist between LA and vasculitis. The purpose of this study was to explore the anti-inflammatory effects of LA, its biological mechanisms, and the effect of LA on TAO.

## 2. Materials and Methods

The experiment was divided into two parts, an *in vitro* experiment on human umbilical vein endothelial cells (HUVECs) and an *in vivo* experiment on rats.

### 2.1. Materials

All chemicals were purchased from commercial sources. HUVECs (CRL-1730) were purchased from ATCC (Manassas, VA, USA). LA (pure standard) was purchased from Shanghai Daoyi Biotechnology Co., Ltd. (Shanghai, China). Monoclonal mouse anti-GAPDH (glyceraldehyde 3-phosphate dehydrogenase) and horseradish peroxidase- (HRP-) conjugated antibody (KC-5G5) were purchased from KangChen Bio-tech Inc. (Shanghai, China). p-Syk (Tyr535/526), ERK (4695S), p-ERK (4370S), p38 (8690S), p-p38 (4511S), JNK (9258S), p-JNK (4668S), and IL-1*β* (12703S) antibodies were purchased from Cell Signaling Technology (Danvers, MA, USA). Antihuman pro-IL-18 (M156-3) and IL-18 (D043-3) antibodies were purchased from Medical & Biological Laboratories (Nagoya, Japan). A Syk enzyme system (V3801) and ADP-Glo kinase assay (V9101) were purchased from Promega (Madison, WI, USA). Human pro-IL-1*β* (MAB6964-SP) antibody was purchased from R&D Systems (Minneapolis, MN, USA). A high-capacity cDNA reverse transcription kit, Lipofectamine 2000, and TRIzol Reagent were purchased from Life Technologies (Carlsbad, CA, USA). An enhanced chemiluminescent (ECL) Immobilon Western Chemiluminescent HRP Substrate (WBKLS0500) was purchased from Millipore (Billerica, MAS, USA). JNK inhibitor (SP600125) and p38 inhibitor (SB203580) were purchased from Selleck Chemicals (Houston, TX, USA).

Sodium laurate was purchased from Sinopharm Chemical Reagent Co., Ltd. (product number: 30168127; Shanghai, China). Medical glues were purchased from Guangzhou Baiyun Medical Adhesive Co., Ltd. (Guangzhou, China).

### 2.2. *In Vitro* Experiments

#### 2.2.1. Cell Culture

HUVEC and HKE293 cells were maintained at 37°C in 5% CO_2_ in Dulbecco's modified Eagle's medium containing 10% fetal bovine serum. The medium was changed every 2 days, and the cells were passaged with trypsin-EDTA. Cells at passages 4–8 were used for the studies.

#### 2.2.2. Monocyte Cell Adhesion Assays

Monocyte U937 cells were grown in Roswell Park Memorial Institute 1640 medium containing 10% fetal calf serum. For the adhesion assays, HUVECs were grown to confluence in 6-well tissue culture plates, after which 100 ng/mL of lipopolysaccharide (LPS) was added for an additional 16 h to induce the expression of VCAM-1, in the presence or absence of LA (0, 5, 25, 50, and 100 *μ*mol/L). For the control, monolayer cells were treated with a mouse antihuman monoclonal antibody (E1/6) against VCAM-1. Adhesion assays were performed by adding 1 mL of the concentrated U937 cells (with a density of 10^6^ cells/mL) to each monolayer under rotating conditions (63 rpm) at 21°C and incubating for 10 min. After nonadherent cells were removed by washing them gently three times with phosphate-buffered saline, the adherent cells in 6-well plates were fixed with 1% paraformaldehyde. The number of adherent cells was determined through counting six different fields by using an ocular grid and a 20x objective. Fields for counting adherent U937 cells were randomly selected at a half-radius distance from the center of the monolayers. Counts were performed in three independent experiments.

#### 2.2.3. In Vitro Syk Activity Assay

Syk activity was measured according to the manufacturer's protocol (Promega, USA). Briefly, Syk (100 ng/*μ*L), a substrate (0.2 *μ*g/*μ*L), adenosine triphosphate (ATP) (10 *μ*mol/L), and LA (0.05–50,000 nmol/L) were diluted in kinase buffer, and 5 *μ*L of inhibitors, 10 *μ*L of an enzyme, and 10 *μ*L of the substrate/ATP mixture were added to each well of a 96-well plate. After incubation at room temperature for 60 min, 25 *μ*L of adenosine diphosphate- (ADP-) Glo™ reagent was added to each well, followed by incubation at room temperature for 40 min. Finally, 10 *μ*L of kinase detection reagent was added to each well, followed by incubation at room temperature for 30 min. Chemiluminescent signal (integration time: 1 s) was recorded using a Varioskan Flash microplate spectrophotometer (Thermo Scientific, USA).

#### 2.2.4. RNA Isolation and Quantitative Polymerase Chain Reaction

Total RNA was extracted from the cells with Invitrogen TRIzol Reagent (Life Technologies) according to the manufacturer's protocol. First-strand complementary DNAs (cDNAs) were synthesized using a high-capacity cDNA reverse transcription kit (Life Technologies). The obtained cDNA was mixed with Maxima SYBR Green quantitative polymerase chain reaction (qPCR) Master Mix (Life Technologies) and gene-specific primers (Shanghai Generay Biotech, China). The sequences of the primers are shown in Table 1. QPCR was performed using a 7500 Fast Real-Time PCR System (Applied Biosystems, USA) according to the manufacturer's instructions. The amplification conditions consisted of an initial 15 min denaturation step at 95°C, followed by 40 cycles of denaturation at 95°C for 15 s, annealing at 55°C for 30 s, and elongation at 72°C for 30 s. The dissociation curves were analyzed to ensure the amplification of a single PCR product. Three independent assays were performed for each primer. The amount of cDNA was calculated for each sample from the standard curve. The relative expression was shown after normalization by the gene expression of GAPDH.

#### 2.2.5. Western Blot

The cells were lysed in ice-cold radioimmunoprecipitation assay buffer (50 mmol/L Tris/HCl, pH 8.0, 150 mmol/L NaCl, 2 mmol/L sodium orthovanadate, 1% Nonidet P-40, 1% sodium deoxycholate, 0.1% sodium dodecyl sulfate, 0.1 mmol/L dithiothreitol, 0.05 mmol/L phenylmethylsulfonyl fluoride, 0.002 mg/mL aprotimin, and 0.002 mg/mL leupeptin) and sonicated using a JY92-2D ultrasonic homogenizer (Ningbo Scientz Biotechnology, Zhejiang, China). Lysates were precleared through centrifugation at 12000 ×g for 10 min at 4°C. Aliquots of the cell lysates (50 or 100 *μ*g of each sample) were resolved on sodium dodecyl sulfate polyacrylamide gel electrophoresis and blotted onto nitrocellulose membranes (Pall China, Shanghai, China). The membrane was blocked in 5% skim milk overnight at 4°C. This was followed by incubation with primary antibodies for 2 h and exposure to a horseradish peroxidase-conjugated secondary antibody for 1 h at room temperature. Visualization was performed using ECL Immobilon Western Chemiluminescent HRP Substrate (Millipore). The membrane was exposed to a high-performance autoradiographic film, (Fuji Film, Tokyo, Japan) and visualized using the ECL Immobilon Western Chemiluminescent HRP Substrate (WBKLS0500; Millipore). A quantitative analysis was performed using Quantity One software. Western blot experiments were performed at least three times.

### 2.3. *In Vivo* Experiment

#### 2.3.1. Animal Ethics Statement and Animals

The animals received care in compliance with the *Guide for the Care and Use of Laboratory Animals* published by the US National Institutes of Health. The animal experiments in this study were approved by the Animal Ethics Committee of Shanghai University of Traditional Chinese Medicine (approval number SZY201602004).

Male Wistar rats were purchased from Vital River Experimental Animal Technology Co., Ltd. (Beijing, China). Rats were housed in cages (three per cage) maintained at constant humidity (65% ± 5%) and temperature (24°C ± 1°C) with a 12-hour light-dark cycle. Rats were allowed ad libitum access to tap water and food throughout the experimental protocols. Thirty-six male Wistar rats were randomly divided into four groups (with nine rats in each): the control group, positive control group (inject of sodium laurate into the femoral artery of the lower extremity to make TAO model), negative control group (inject of saline solution into the femoral artery of the lower extremity), and TAO and LA group. Rats in the TAO and LA group were treated with LA for 16 days (20 mg/kg/day, 2 days before surgery, and 2 weeks after surgery). By contrast, rats in the control group, positive control group, and negative control group were given the adjuvant (0.30% CMC-Na) only. Two weeks after surgery, rats were anesthetized using an intraperitoneal injection of sodium pentobarbital at a dose of 45 mg/kg. Blood was taken from the abdominal aorta, and serum was separated; the right lower limb aorta was isolated and then stored at −80°C.

#### 2.3.2. Rat TAO Model

Before surgery, the baseline blood flow was measured through laser speckle flowmetry with the PeriCam PSI System (Perimed AB, Stockholm, Sweden). An intraperitoneal injection of 3% sodium pentobarbital (45 mg/kg) was administered to anesthetize each rat; each injected rat was then fixed on a plate. The right groin was taken as the surgical position, which was shaved and disinfected with iodophor. The midpoint of the groin was incised approximately 1.5 cm, and the subcutaneous tissue and muscle tissue were bluntly dissected, exposing the femoral artery. Then an artery clip was used to block the blood flow of the proximal heart end to separate the superficial part of the abdominal artery (approximately 0.5 cm in length), and 10 mg/mL sodium laurate 0.2 mL was injected to the distal direction [13–15]; further, medical glue was used to close the puncture point. Next, the artery clip was opened 1 min after surgery, and after confirming that there was no bleeding, sutures were applied to the skin. The same surgical method was used for the negative control group, but 0.2 mL of physiological saline was injected instead of sodium laurate. After the operation, the rats were placed on a thermal pad for 10 min to recover body temperature. The effect of the operation was confirmed using laser speckle flowmetry 30 min after surgery. The blood flow was then monitored at 2, 5, 8, 11, and 14 days.

#### 2.3.3. Enzyme-Linked Immunosorbent Assay

An enzyme-linked immunosorbent assay (ELISA) was performed using a rat IL-1*β* ELISA kit (RLB00, R&D Systems, USA), rat interleukin-6 (ERC003.96, Xinbosheng Biological Technology Co., Ltd., Shenzhen, China), and C-reactive protein (CRP) ELISA assay kits (H126, Nanjing Jiancheng Bioengineering Institute, Nanjing, China); all kits were used according to the manufacturers' instructions.

### 2.4. Statistical Analysis

Data were analyzed using GraphPad Prism 5 or SPSS 15.0 software. A paired *t*-test was used to compare the two groups, and a one-way analysis of variance was used for comparisons within groups. Data are expressed as mean ± standard deviation (SD), and *p* < 0.05 was considered significant.

## 3. Results

### 3.1. In Vitro Results

To test the anti-inflammatory effects of LA, real-time qPCR was employed to detect the messenger RNA (mRNA) expression of different proinflammatory genes. After the HUVECs were stimulated using LPS for 6 h, the mRNA expressions of IL-1*β*, TNF-*α*, IL-32*β*, MCP-1, CCL-2, and VCAM-1 were increased by 1.13- to 1.83-fold compared to control groups. However, pretreatment with LA (50 *μ*mol/L) for 1 hour prior to LPS stimulation almost completely blocked the enhanced mRNA expressions near or below the baseline (Figure 1(b)). The effects of LA on monocyte adhesion to endothelial cells mediated by VCAM-1 were examined. As presented in Figure 1(c), after incubation of the HUVECs with LPS (16 h), adherent U937 monocytes markedly increased to 74.0 ± 3.9 cells/mm^2^, which was almost completely blocked by treatment with a mouse antihuman monoclonal E1/6 antibody against VCAM-1. Pretreatment with various doses of LA (5–100 *μ*mol/L) inhibited U937 monocyte adhesion in a dose-dependent manner. Monocyte adhesion to the HUVECs significantly decreased after LA treatment from 34.5 ± 6.4 cells/mm^2^ (5 *μ*mol/L, *p* < 0.05) to 20.0 ± 2.8 cells/mm^2^ (100 *μ*mol/L, *p* < 0.005).

**Figure 2:**
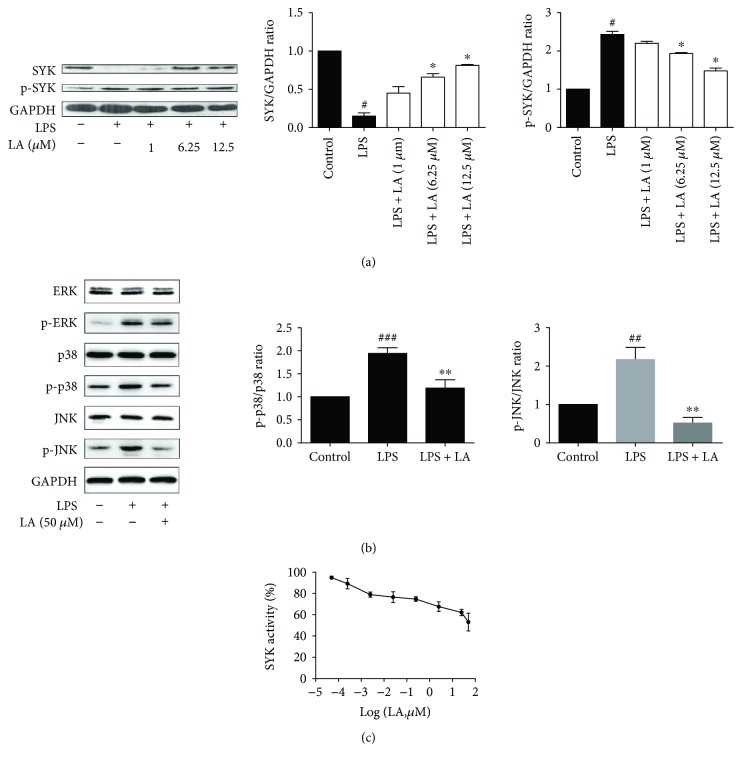
LA inhibited Syk and p38/JNK activity. HUVECs were pretreated in the presence of various doses of LA for 1 h and stimulated with LPS for 30 min. A Western blotting analysis was performed to evaluate the levels of phosphorylated Syk and total Syk (a). HUVECs were treated with 50 *μ*M of LA for 1 h before LPS stimulation for 15 min, and then the total proteins of cells were extracted. The activation of the MAPK (b) pathways was detected using Western blotting with specific antibodies. Syk activity was analyzed using an *in vitro* kinase assay through recombinant Syk obtained from Promega. The assay (ADP-Glo kinase assay) was performed to measure luminescent signals by using Ultra-Glo Luciferase; its ADP from a kinase reaction is converted into ATP converted into light. Kinase assays were conducted in the presence of various doses of LA for 30 min. The results were evaluated using Syk activity (c). Data represent the mean ± SD of at least three independent experiments, and each experiment was performed in triplicate. ^#^*p* < 0.05^##^*p* < 0.01 and ^###^*p* < 0.001 versus control alone; ^∗^*p* < 0.05^∗∗^*p* < 0.01 versus LPS alone.

Next, the effects of LA on Syk activity were examined. The results revealed that LPS induced Syk phosphorylation and nonphosphorylated Syk depletion, whereas increased doses of LA inhibited Syk phosphorylation (Figure 2(a)). In order to confirm whether LA mediated the mitogen-activated protein kinase (MAPK) pathway, activation of ERK, p38, and JNK was examined by Western blotting. The results show that LA inhibited the activation of p38 and JNK in LPS-stimulated HUVECs. Compare with the LPS group, phosphorylation levels of p38 and JNK in LA pretreated cells were reduced by 1.74 and 4.36 times, respectively (Figure 2(b)). To test whether LA directly blocked Syk activity, Syk kinase assay kit was used to determine the inhibitory effects of LA; the results revealed that LA inhibited Syk activity in a dose-dependent manner with an EC50 value 0.129 *μ*mol/L (Figure 2(c)).

**Figure 3:**
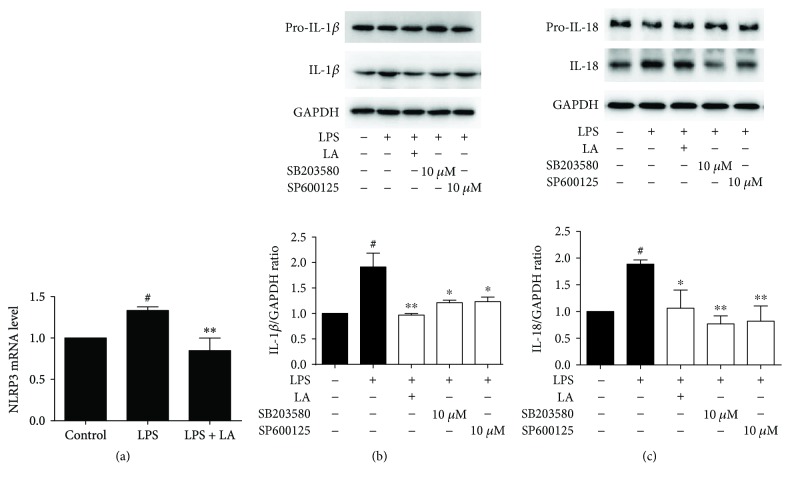
LA suppressed NLRP3 gene and downstream related protein expression. HUVECs were treated with 50 *μ*M of LA for 1 h, and then LPS was stimulated for 24 h. The NLRP3 mRNA level (a) was analyzed using qRT-PCR. LA and p38/JNK inhibitors (SB203580 and SP600125) were treated with HUVECs for 60 min before LPS stimulation for 24 h. NLRP3-associated proteins IL-1*β* and IL-18 were detected using immunoblotting (b)(c). Data represent the mean ± SD of at least three independent experiments, and each experiment was performed in triplicate. ^#^*p* < 0.05 versus control; ^∗^*p* < 0.05^∗∗^*p* < 0.01 versus LPS.

To observe the effect of LA on NLRP3 (a downstream gene of Syk), the expression of NLRP3 was detected through RT-PCR. After HUVEC cells were stimulated by LPS for 6 h, the mRNA expressions of NLRP3 was increased by 1.33-fold compared to the control group. However, pretreatment with LA for 1 h prior to LPS stimulation the expression of NLRP3 is 0.84 times lower than control group. The results indicated that LA could downregulate NLRP3 gene expression (Figure 3(a)). The NLRP3 inflammasome was responsible for the expression of mature IL-1*β* and IL-18. Western blotting was used to elucidate the role of LA in LPS-induced IL-1*β* and IL-18 release. The results showed that LA substantially blocked the LPS-induced upregulation of IL-1*β* and IL-18 compared with treatment with LPS alone. This effect was similar to the JNK and p38 inhibitors (Figures 3(b) and 3(c)). These results indicated that LA might reduce the promotion of the NLRP3 inflammasome to inhibit inflammation.

### 3.2. In Vivo Results

**Figure 4:**
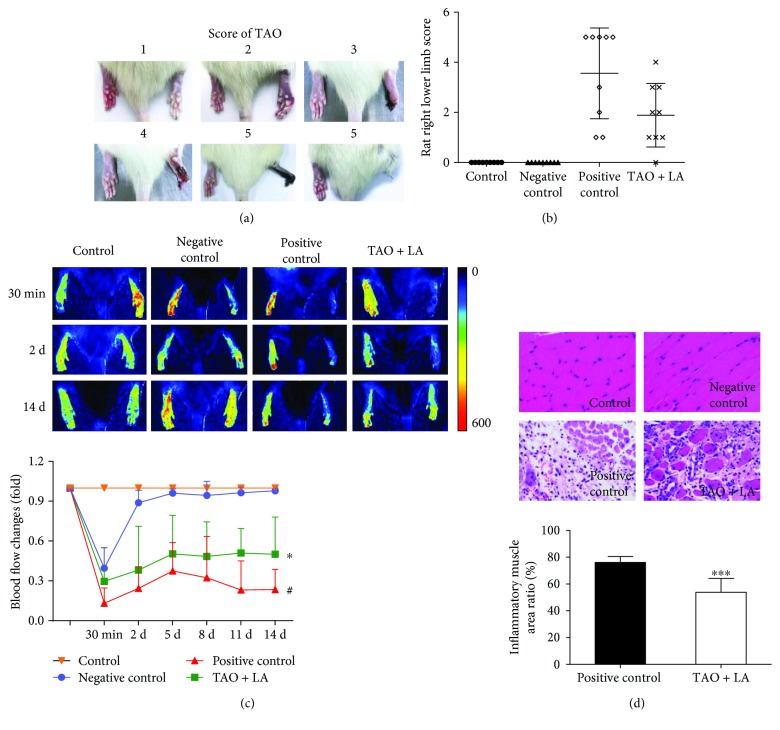
LA reduced the damage to the rats' lower limbs in the TAO model. A right lower extremity arterial injection of sodium laurate 10 mg/mL 0.2 mL was used in the positive control group, and 0.9% physiological saline 0.2 mL femoral artery injection was used in the negative control group. The effect of the operation was confirmed using laser speckle flowmetry 30 min after surgery. The rats in the control group and positive and negative control groups were given the adjuvant (CMC-Na) only, whereas the LA group received 20 mg/kg/d for 2 weeks, which started to gavage 2 d before surgery. One week after surgery, various degrees of gangrene were observed in the rats' lower right limbs, as shown in (a). The score of the right lower limb of each group was assessed according to the TAO model score standard (b). The blood flow of the lower limbs in each group was measured using laser speckle flowmetry (c). Inflammatory necrosis of the right leg muscle was observed using HE-stained slices (400^∗^) (d). Data are presented as mean ± SD. ^#^*p* < 0.05 versus negative control group; ^∗^*p* < 0.05 and ^∗∗∗^*p* < 0.001 versus positive control group.

After injection with sodium laurate, different degrees of ischemic necrosis occurred in the right lower limbs of rats in the positive control group and the TAO + LA group (Figure 4(a)). According to the vasculitis model score criteria [16], the score of the positive control group was significantly higher than that of the TAO + LA group after surgery for 2 weeks (Figure 4(b)). Then, laser speckle flowmetry was used to determine the blood flow of the lower extremities of the rats in each group. The blood flow of the negative control group was higher than that of the positive control group and TAO + LA group after the operation for 30 min. The TAO + LA group was treated intragastrically 2 days before surgery; the blood flow of the TAO + LA group was higher than that of the positive control group. The blood flow in the right legs of the negative control group returned to normal 2 weeks after the operation, whereas the blood flow in the right legs of the TAO + LA group and positive control group was lower than that of the control group, and the blood flow intensity in the TAO + LA group was higher than that in the positive control group (Figure 4(c)). Two weeks after the operation, the right lower extremity muscles of each group were examined using hematoxylin and eosin (HE) staining. The degree of muscular inflammatory necrosis in the positive control group was significantly higher than in the TAO + LA group (Figure 4(d)).

**Figure 5:**
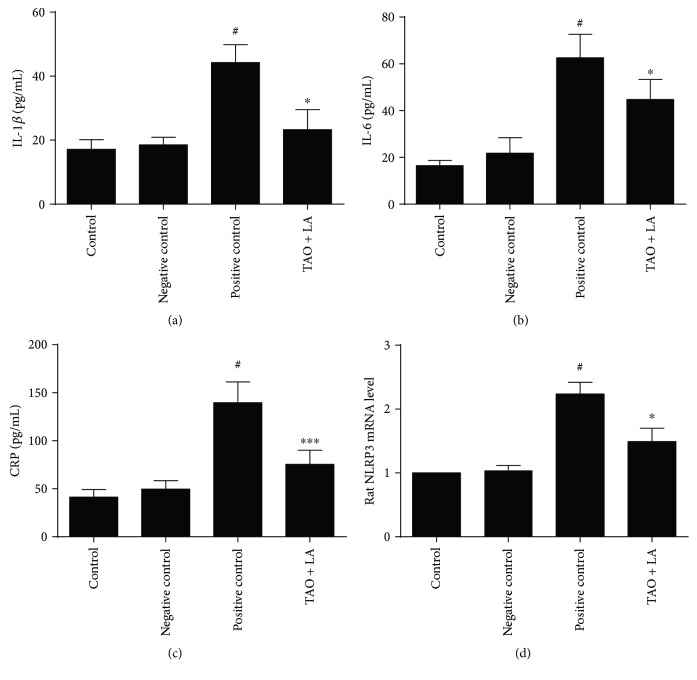
LA reduced the rats' inflammatory factor levels. Wistar rat serum IL-1*β*/IL-6/CRP content was detected using an ELISA (a–c). Lower limb arterial NLRP3 mRNA levels in rats (d). ^#^*p* < 0.05 versus negative control group; ^∗^*p* < 0.05 and ^∗∗∗^*p* < 0.001 versus positive control group.

The levels of inflammatory cytokines were detected through ELISA. The serum IL-1*β*, IL-6, and CRP in the positive control group was higher than those in the control group and negative control group. Compared with the positive control group, the serum IL-1*β*, IL-6, and CRP levels were significantly decreased in the TAO + LA group rats (Figures 5(a)–5(c)). LA reduced the expression of NLRP3 in the rats' lower limb arteries (Figure 5(d)).

## 4. Discussion

Liu et al. proposed that inflammation and the immune system play central roles in TAO pathogenesis [17]. LPS is a component of Gram-negative bacteria involved in the transcription of numerous proinflammatory genes. LPS is a commonly used stimulator of inflammation models and is often used for *in vitro* mechanism studies of TAO [18, 19]. Toll-like receptor 4 (TLR4) is activated by LPS. TLR4 can induce the production of proinflammatory mediators; this effect of TLR4 can be applied to eradicate bacteria. CD14 transfers LPS to the TLR4/MD-2 complex, which dimerizes and triggers the MyD88- and TRIF-dependent production of proinflammatory cytokines and type I interferons [20]. LPS-activated TRL4 activates the NFkappaB pathway and all three MAPK pathways (ERK, JNK/SAPK, and p38). LPS also stimulates NLRP3 inflammasomes, the best-characterized NLR molecules, which induces the release of mature IL-1*β* and IL-18 [21].

**Figure 6:**
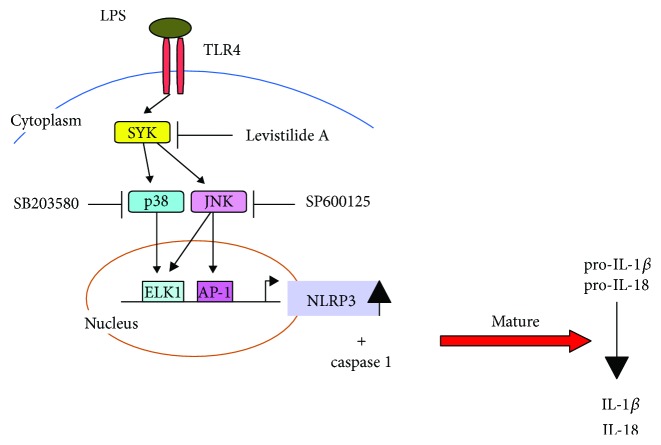
LA anti-inflammatory mechanism diagram. LA inhibited the activity of p38/JNK by decreasing Syk phosphorylation, thereby inhibiting the downstream NLRP3 inflammasome gene expression and the secretion of its related proteins (IL-1*β* and IL-18).

The study revealed that LA directly inhibited Syk activity and Syk phosphorylation. Syk plays a crucial role in adaptive immune receptor signaling and numerous biological processes, including cellular adhesion, innate immune recognition, osteoclast maturation, platelet activation, and vascular development [22]. Syk activity is induced by LPS, TNF-*α*, IL-1, thrombin, C-response protein, minimally oxidized LDL, and high glucose levels [23–29]. It is transiently recruited to a complex containing MyD88, TLR4, and IRAK-1 upon LPS stimulation and disassociates over time [24]. Notably, the kinetics of TLR4 tyrosine phosphorylation coincides with an early wave of Syk tyrosine phosphorylation. Syk is also required for cytokine production with MyD88 adaptor protein pathways [30]. Numerous studies have demonstrated that Syk-mediated mitogen-activated protein kinases play a vital role in the expression of inflammatory genes [31–33]. Syk mediates NLRP3 inflammasome activation [34–36]. Therefore, one can infer that by inhibiting the phosphorylation of Syk, LA inhibits the activity of p38/JNK and reduces both the expression of NLRP3 and the secretion of related proteins IL-1*β* and IL-18 (Figure 6).

TAO is a rare disease of unknown etiology, and despite its discovery more than a century ago, little progress has been made in determining its mechanism and suitable treatments. In a thrombohemorrhagic vasculitis model, the mouse vessel wall Syk tyrosine kinase signaling pathway was triggered. This led to neutrophil elastase release, which caused hemorrhage, fibrin deposition, and thrombosis [37]. Sun and colleagues proposed potential mechanisms from a genetic and immunoreactive perspective for its inception and revealed that the pathogenesis of TAO is due to a type of gene polymorphism that leads to immunological inflammatory vasculitis, which is highly linked to HVECs and the TLR-MyD88-NFkB pathway [38]. Neutrophil aggregation and their interaction with the endothelium and platelets as well as neutrophil-derived microparticles promote the generation of occlusive thrombi, fibrin accumulation, and hemorrhage [39–41]. Monocyte aggregation contributes to thrombus formation. Studies have revealed that macrophages infiltrated the internal lamina and that VCAM-1 and ICAM-1 were expressed in the vascular endothelium of TAO lesions [42, 43]. The endothelial cell experiment revealed that LA inhibited the adhesion of monocytes to endothelial cells. Animal studies have shown that LA reduces the degree of necrosis of the lower extremities in TAO rats, improves blood supply, and reduces the expression of NLRP3 in blood vessels. LA may achieve these benefits by reducing Syk activity and inflammatory damage to endothelial cells. This hypothesis has not yet been validated by *in vivo* experiments; this lack of confirmation may be one of the limits of the present study. Generalized functional arterial disorder in patients with TAO with functional deterioration of the brachial artery could be related to increased levels of various inflammatory markers [3]. The serum levels of IL-6 and IL-8 significantly decreased after TAO was treated with conventional therapy [44]. This was consistent with the decreases in serum IL-6, IL-8, and CRP after treatment of TAO rats with LA.

Few pharmacological and surgical options are available to treat TAO other than TAO therapy in addition to the discontinuation of cigarette smoking [45]. The frequent use of high-potency vasodilators, such as iloprost, bosentan, sildenafil, or alprostadil, relieves symptoms and reduces the risk of amputation. Although two randomized trials have demonstrated the efficacy of intravenous iloprost, no oral drug has been demonstrated to be effective in treating TAO. This study provides a biological mechanism for the anti-inflammatory effects of LA and a theoretical basis for the development of a drug for treating TAO.

## 5. Conclusions

LA displayed anti-inflammatory properties; some potential mechanisms involved the Syk-p38/JNK signal pathway. The results of this study indicate that LA can treat vascular diseases associated with inflammation such as TAO.

## Figures and Tables

**Table 1:** Quantitative RT-PCR primer sequences.

Gene	Direction	Primer
TNF-*α*	Forward	CAGAGGGAAGAGTTCCCCAG
	Reverse	CCTTGGTCTGGTAGGAGACG
IL-1*β*	Forward	AAACAGATGAAGTGCTCCTTCCAGG
	Reverse	TGGAGAACACCACTTGTTGCTCCA
VCAM-1	Forward	TTGCTCAGATTGGTGACTCCGTCT
	Reverse	TTCGTCACCTTCCCATTCAGTGGA
NLRP3	Forward	TTCGGAGATTGTGGTTGGG
	Reverse	CAGGTAAAGGTGCGTGAGATT
IL-32*β*	Forward	GAGTTTCTGCTGCTCTCTGTCA
	Reverse	ATTTTGAGGATTGGGGTTCAG
eNOS	Forward	TCACCGCTACAACATCCT
	Reverse	CCTTCTGCTCATTCTCCA
GAPDH	Forward	AGAAGGCTGGGGCTCATTTG
	Reverse	AGGGGCCATCCACAGTCTTC
NLRP3 (rat)	Forward	TGAAGAGTGTGATCTGCGGAAAC
	Reverse	GAAAGTCATGTGGCTGAAGCTGT
GAPDH (rat)	Forward	CAACGGGAAACCCATCACCA
	Reverse	ACGCCAGTAGACTCCACGACAT

## Data Availability

The data used to support the findings of this study are available from the corresponding author upon request.

## Abbreviations

GAPDH: Glyceraldehyde 3-phosphate dehydrogenase
HEK293: Human embryonic kidney cells 293
HRP: Horseradish peroxidase
HUVECs: Human umbilical vein endothelial cells
IL: Interleukin
LA: Levistilide A
LPS: Lipopolysaccharide
MCP-1: Monocyte chemotactic protein-1
PBS: Phosphate-buffered saline
Syk: Spleen tyrosine kinase
U937: Human leukemic monocyte lymphoma cell line
VCAM: Vascular cell adhesion molecule
TAO: Thromboangiitis obliterans.
